# A Case of Facultative Polygyny in an Enigmatic Monogamous Species, the European Nightjar (*Caprimulgus europaeus*)

**DOI:** 10.1002/ece3.70366

**Published:** 2024-10-17

**Authors:** Ruben Evens, Michiel Lathouwers, Jitse Creemers, Eddy Ulenaers, Marcel Eens, Bart Kempenaers

**Affiliations:** ^1^ Department of Biology, Behavioural Ecology and Ecophysiology Group University of Antwerp Antwerp Belgium; ^2^ Department of Ornithology Max Planck Institute for Biological Intelligence Eberhard‐Gwinner‐Straße Germany; ^3^ Earth and Life Institute, Terrestrial Ecology and Biodiversity Conservation Group Université Catholique de Louvain Louvain‐la‐Neuve Belgium; ^4^ Centre for Environmental Sciences, Research Group Zoology, Biodiversity and Toxicology Hasselt University Diepenbeek Belgium; ^5^ Department of Geography University of Namur Namur Belgium; ^6^ Agentschap Natuur en Bos, Regio Noord‐Limburg Brussels Belgium

**Keywords:** accelerometer data, breeding ecology, crepuscular, GPS‐tracking, nightjars, reproduction, space use, territoriality

## Abstract

In many socially monogamous bird species with biparental care, occasional social polygyny has been detected. We provide information about a case of facultative polygyny in the European Nightjar (*Caprimulgus europaeus*). The male nightjar (I96) formed a pair with two females (I95: the presumed primary female with whom he already bred since 2018; M042: the presumed secondary female, an inexperienced yearling). GPS and accelerometer data demonstrate how the male only sang in proximity of the primary nest, while assisting both females during incubation, as well as during the nestling period. When the male came to the nest, the primary and/or secondary female went foraging, but the secondary female received less assistance during incubation than the primary female, and her eggs were often left unattended. However, once the chicks of the secondary female hatched, male assistance suddenly increased, presumably at a cost to the primary female. Being only the second record of social polygyny in the European Nightjar, we do not have a direct explication for the occurrence of this polygynous event. We note that male density at the study site was lower than that observed in previous seasons. The male may have taken over the female that was initially paired to a neighbouring territory holder that then died. Alternatively, the inexperienced female might have mated with an already paired male, either because she was not aware of the mating status of the male, or because she could not find an unpaired male, or because mating with this paired male was better than mating with another unpaired male. In any case, the breeding ecology and mating behaviour of this crepuscular bird species remains little understood.

## Graphical abstract

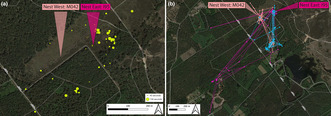

## 1 Introduction

In many socially monogamous bird species with biparental care, occasional social polygyny has been detected (Cockburn 2006; Lack 1968). Polygyny can occur opportunistically, for example if a pair is joined by a female that recently lost its mate (take‐over polygyny). Alternatively, social polygyny can be a strategy when males invest in attracting additional mates, for example by defending another territory (Slagsvold et al. 1988). In case of facultative‐polygynous species with biparental care, a conflict of interest may exist between the sexes because males may increase their fitness by attracting additional females (Arnqvist and Rowe 2005) while polygyny can be costly to females if male assistance is reduced (Lubjuhn et al. 2000; Moreno et al. 2002). However, due to paternity loss, socially polygynous males do not always have higher reproductive success (Schlicht and Kempenaers 2021).

Prospecting females may choose a partner based on several factors, including variation in male or territory quality, prior experience with the male (Kempenaers 1994), the availability of nest sites, or the local availability of unpaired males (i.e., the local sex ratio) (Canal et al. 2021). In migratory species, late‐arriving females may have less time to assess local male quality or to find the few remaining unpaired males (Slagsvold et al. 1988; Sandell 1998). Females may also be deceived by males if they are unaware that the courting male is already mated (deception hypothesis; Alatalo et al. 1981). Alternatively, females may deliberately select an already mated male when its territory or individual quality compensates for the expected costs of the reduction in male parental care (polygyny threshold model; Orians 1969) or the future attractiveness of their young exceeds the costs in terms of other fitness components (sexy son hypothesis; Weatherhead and Robertson 1979).

The occurrence of facultative social polygyny in otherwise socially monogamous species is likely explained by case‐specific circumstances and decisions made by the involved individuals (Walker and Marzluff 2017). Here, we describe a case of facultative polygyny in the European Nightjar *Caprimulgus europaeus* (hereafter nightjar) that was discovered based on GPS‐data and accelerometer data. Nightjars belong to the familiy *Caprimulgidae*, comprising approximately 97 species whose breeding ecology is generally poorly understood. Although for many species the available information is limited, most species are presumably socially monogamous with biparental care. Only three species exhibit extreme plumage dimorphism (i.e., male ornaments, such as elongated feathers that are not present in females) and male emancipation from parental care, suggesting a socially polygynous mating system (Standard‐winged Nightjar *Caprimulgus longipennis*, Pennant‐winged Nightjar *Caprimulgus vexillarius* and White‐winged Nightjar *Eleothreptus candicans*) (Pople 2014; Holyoak 2001).

The nightjar is a long‐distance migratory species and is considered socially monogamous. As has been reported in the related Red‐necked Nightjar *Caprimulgus ruficollis* (Sàez and Camacho 2016), males perform mate guarding in the days and nights preceding egg laying, that is, they spend much of their time close to their female partner (R. Evens and M. Lathouwers, personal observations), presumably to reduce the risk of paternity loss. Most males participate in incubation, brooding and feeding offspring and they may have two clutches per season. The male then often cares alone for the chicks from the first brood after they are 10 days old, while the female initiates the second clutch (Holyoak 2001). In such presumed socially monogamous pairs with two broods, additional ‘extra‐pair’ males have been observed that assist the female in raising the second brood (Cresswell and Alexander 1990; Padget et al. 2019). It is unknown whether these ‘extra‐pair’ males also sired offspring in that brood, or whether they formed a social bond with the female. Mate switching may also occur both between broods within a single season and between seasons (Cresswell and Alexander 1990). A single case of polyterritorial polygyny has previously been described (Odder Jensen 2013). This study reported on a radio‐tagged male nightjar that defended two territories approximately 5 km apart, but did not determine the location of the nests. During the breeding season, the male visited each of his territories with a 1‐day interval. In July, the male remained in his presumed primary territory for 3 weeks after which he was observed flying with a female and two recently fledged juveniles. In his presumed secondary territory, he was observed flying and communicating with a female only on a few occasions.

Our aim here is twofold. First, we provide information about a case of facultative polygyny in a single male territory to identify factors that may contribute to individual variation in mating decisions in the nightjar. Second, we describe how the polygynous male allocated parental care to the two broods.

## 2 Methods and Results

### 2.1 Field Observations

**FIGURE 1:**
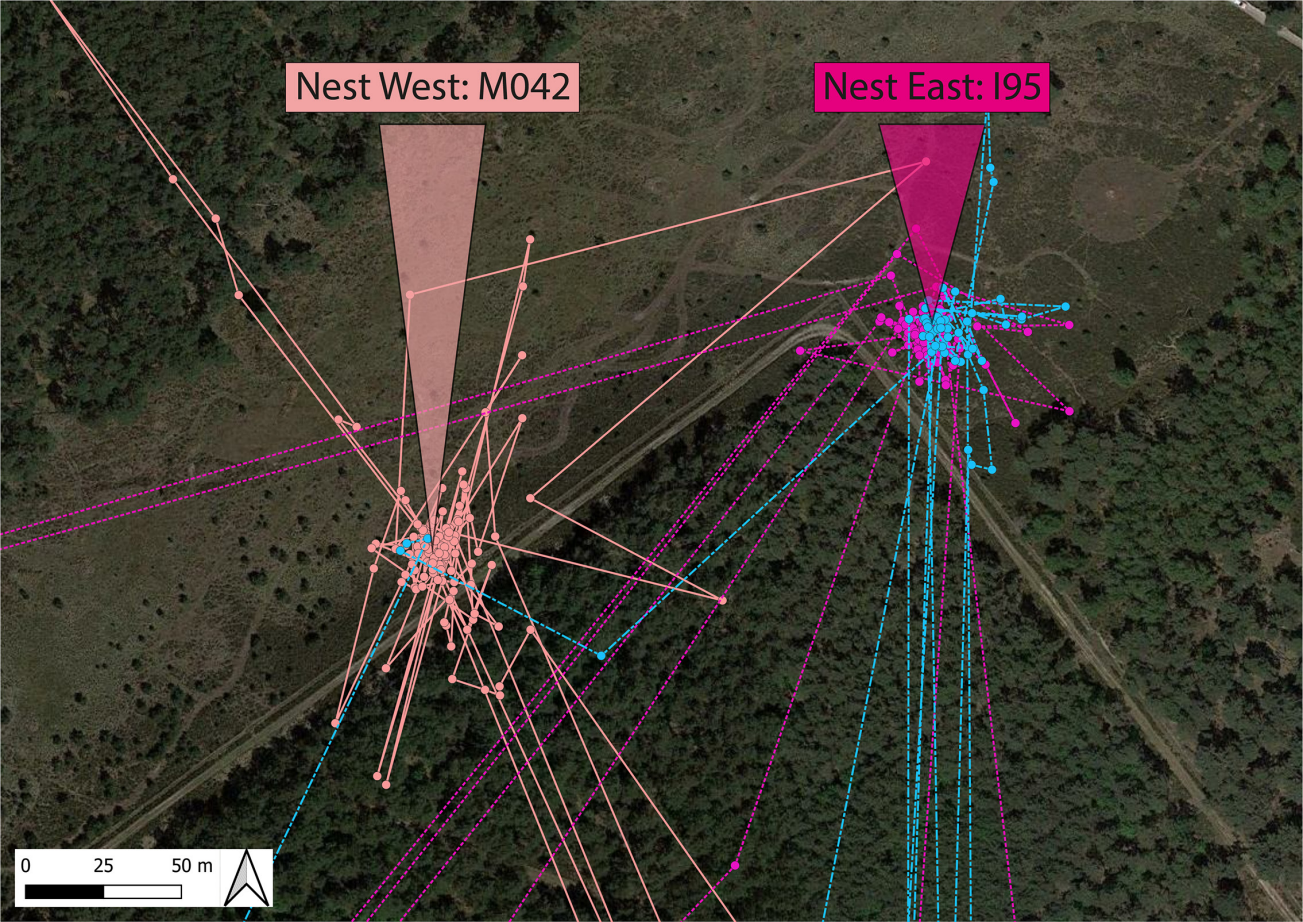
Location of the two nests discovered on 29 June 2022 and an example of GPS‐data demonstrating the nest attendance of male I96 (blue). The eastern nest (2 eggs, hatching: 6 July) is the nest of female I95, the presumed primary female with whom male I96 bred since 2018. The western nest (2 eggs, hatched 11 July) is that of female M042, the presumed secondary female of the same male. GPS‐data show the attendance of male I96 (blue) to the nest of primary female I95 (dark pink) and secondary female M042 (light pink) on 5 July. The male made a single, brief visit to the nest of the secondary female M042 and more frequent visits with longer attendance to the nest of the primary female I95. GPS‐sampling interval = 3 min.

On 29 June 2022, we searched for nightjar nests in a ~5 ha heathland in Grenspark Kalmthout (51.38° N, 4.42° E; Belgium) that consists of typical breeding habitat comprising open heathland with sparse ±3 m high trees (Evens et al. 2017). The site held two or three territories in previous years. We discovered two nests that were 150 m apart (Figure 1), a typical distance between neighbouring territories (R. Evens and M. Lathouwers, personal observations). We therefore assumed that the two nests were from two pairs occupying neighbouring territories, also because each nest was close to a nesting location from previous years. The eastern and western nest both contained two eggs that hatched on 6 and 11 July, respectively.

On the same day, starting 1 h before dusk, we attempted to capture the presumed nightjar pair at the western nest (Figure 1) by placing two ultra‐fine mist nets (Ecotone, 15 × 3 m) parallel to each other left and right of the nest. This set‐up avoids disturbing the incubating female and enables to capture both the female when she leaves the nest at dusk, and the territorial male when he arrives to relieve the female from the nest. We failed to capture the female and we did not detect a male that attended this nest.

On 4 July 2022, we attempted to capture the presumed pairs at both nests, using the same method. Around dusk, we captured the pair of the eastern nest: the adult (> 2CY) male I96 and the adult female I95, who already bred as a pair in the same territory since 2018 (both individuals were captured on a nest in the same territory in 2018, 2019 and 2022). Around the same time, we captured the yearling (2CY) female M042 at the western nest. Again, we did not detect a male at the western nest. Similarly, during a third capture attempt at that nest on 8 July 2022, we did not observe a male at the nest.

**FIGURE 2:**
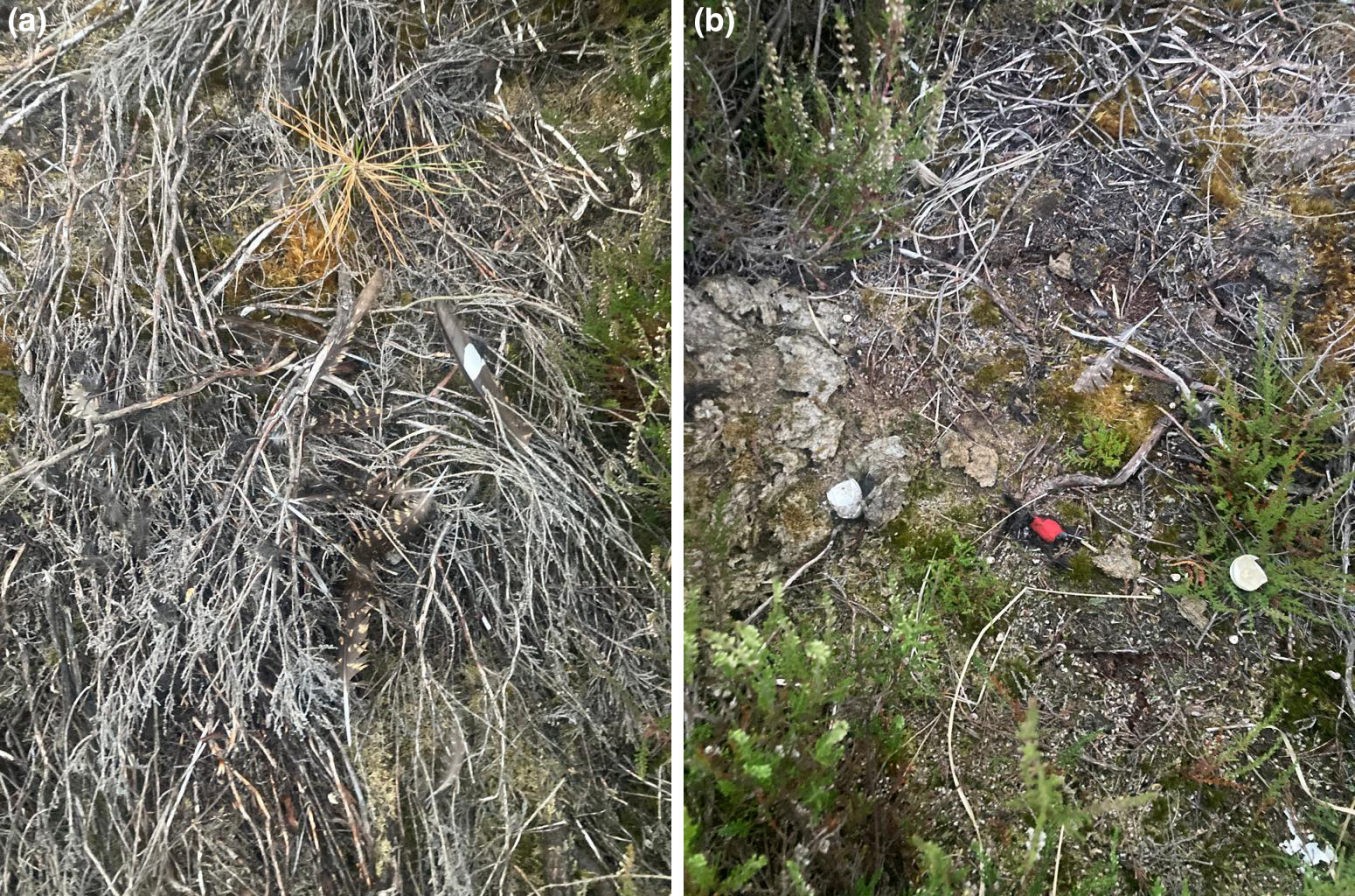
Photos of (a) the remains of male I96 and (b) the recently hatched chicks (chick‐droppings and egg shell) in the nest of secondary female M042. Also note the presence of the logger of male I96 near the nest (A; red).

On 20 July 2022, we visited both nests and found them predated. At the eastern nest, we recovered the GPS logger of female I95. At the western nest, we recovered the GPS logger of female M042 and the remains of male I96, including its logger (Figure 2). We also recovered two body feathers of an Eagle Owl (*Bubo bubo*), the presumed predator.

### 2.2 Information From Data Loggers

All three individuals had been fitted with a custom‐designed combination of an activity logger (1.4 g Technosmart Axy5), a radio tag (0.4 g; Biotrack Ltd.) and a GPS logger (1.8 g; Pathtrack Ltd.), attached to the base of the tail with a simple ‘drop‐off’ mechanism (Evens et al. 2018). The combined tag weighed approximately 5% of the body mass of the tagged birds (male I96: 69 g, female I95: 74 g, female M042: 83 g). We programmed the GPS‐loggers to fix positions at 3‐min intervals from before sunset (9 PM, UTC + 2) until after sunrise (6 AM, UTC + 2). To obtain synchronous activity data, we programmed activity loggers to measure acceleration continuously at 25 Hz (*g*, in the *X*‐, *Y*‐ and *Z*‐axis) within the same timeframe. This allowed the logging of the individuals' activity for approximately 100 h. For further analysis, we calculated dynamic body acceleration by smoothing the activity data using a running mean for 2‐s intervals and subtracting the smoothed data from the unsmoothed data to remove the static acceleration (i.e., acceleration resulting from the body angle with respect to the earth's gravity). The loggers of female I95 and M042 contained three nights of relevant GPS data, but due to a software malfunctioning we could not use the accelerometer data of these two loggers. The logger of male I96 contained 11 nights of GPS data and 10 days of accelerometer data.

**FIGURE 3:**
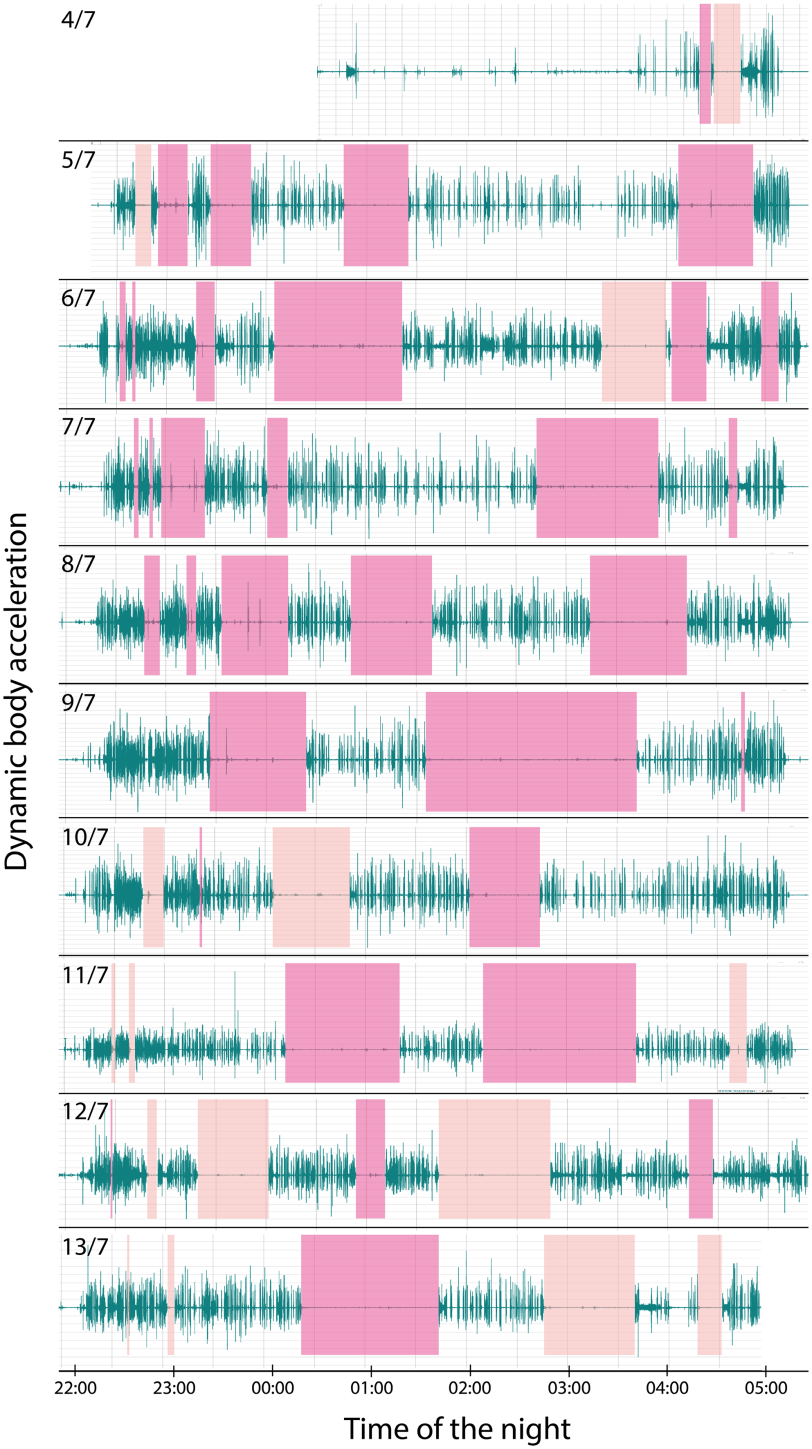
Daily dynamic body acceleration of male I96 between 4 and 13 July 2022. Periods of inactivity (i.e., no flight or song activity) where the male attended one of the nests to incubate, brood or feed are indicated by pink boxes (dark: Visits to the nest of primary female I95, light: Visits to the nest of secondary female M042).

**FIGURE 4:**
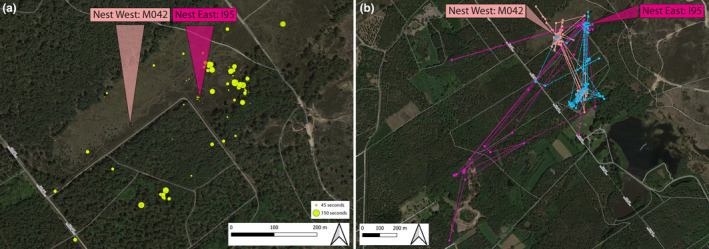
Song activity of male I96 between 4 and 13 July 2022 and GPS‐data demonstrating the location of foraging sites of the polygynous trio. (a) Shown are GPS‐data for which song activity was registered based on the accelerometer data. Circle size indicates the duration of song activity in seconds. The data show that male I96 sings in proximity of the nest of the primary female (I95), at presumed fixed song posts in his territory, whereas there is no song activity close to the secondary nest (female M042). The cluster of song activity in the central lower part of the figure represents the roost of the male, where he sings around sunset and sunrise. (b) GPS‐data from 5 July 2022, demonstrating the location of foraging sites of male I96 (blue), primary female I95 (dark pink) and secondary female M042 (light pink) relative to their nesting sites (arrows). The data show that the foraging sites of secondary female M042 and male I96 overlap to some extent, while primary female I95 forages in a separate location further (south‐)west. GPS‐sampling interval = 3 min.

We estimated the investment of the male in parental care at the two nests as follows. First, we used the GPS‐data to define periods when male I96 was near (between 0 and 25 m, taking possible GPS‐error into account; Evens et al. 2018) each of the nests (Figure 1). Second, we used the accelerometer data to determine periods of inactivity (i.e., not flying or singing), assuming that the male was either incubating the eggs or brooding the chicks (Figure 3). Note that with this procedure we might have somewhat underestimated the true presence at the nests, because nest visits shorter than the 3‐min GPS sampling interval were not included. Third, we used the accelerometer data to quantify the song activity of the male (Eisenring et al. 2022) to determine where the male sang with respect to the location of the two nest sites (Figure 4A).

The GPS and accelerometer data showed that male I96 only sang in proximity of the eastern nest, reflecting the presumed song posts from the previous year. However, the male visited the nests of both females during the 10‐day tracking period, although the frequency and duration differed between nests (Figures 1 and 3; Table 1). The number of visits to the eastern nest (female I95; *n* = 34) was higher than the number of visits to the western nest (female M042; *n* = 19). Moreover, male visits to the eastern nest lasted longer (paired *t*‐test: *t* = 2.73, *p* = 0.009; I95: 30 min ± 1 min (mean ± SE); M042: 19 min ± 24 s). During the first six tracking nights (4–9 July; Figure 3) the accelerometer data suggest that the male made short visits at dusk and dawn to the chicks of I95, and brooded the chicks for longer periods at night. During the same period, the male only visited the nest of M042 once per night (*n* = 3 nights) or not at all (*n* = 3 nights). The acceleration data suggest that the male incubated the eggs at the nest of M042 twice during this period (on 5 and 6 July 2022; Figure 3). On 11 July 2022, the number of short dusk visits to M042 increased (Figure 3), and on 12 and 13 July 2022, the male also showed prolonged nocturnal visits at the western nest, suggesting that he started feeding and brooding the recently hatched chicks at this nest too. The time spent at the eastern nest, where offspring were approximately 6 days old, decreased during this period.

**TABLE 1:** Overview of data on male I96's nest attendance at the nest of primary female I95 and at the nest of secondary female M042.

Nest I95					Nest M042				
Day	Visit	Start	Stop	Duration	Day	Visit	Start	Stop	Duration
05‐07‐22	1	2:19	2:26	0:03	05‐07‐22	1	2:28	2:44	0:16
05‐07‐22	1	20:52	21:13	0:21	05‐07‐22	1	20:40	20:46	0:06
05‐07‐22	2	21:25	21:49	0:24					
05‐07‐22	3	22:43	23:22	0:39					
06‐07‐22	4	2:16	2:49	0:33					
06‐07‐22	1	20:32	20:35	0:03	07‐07‐22	1	1:22	1:43	0:21
06‐07‐22	2	20:40	20:41	0:01					
06‐07‐22	3	21:18	21:29	0:11					
06‐07‐22	4	22:05	23:21	1:16					
07‐07‐22	5	2:03	2:25	0:22					
07‐07‐22	6	2:58	3:08	0:10					
07‐07‐22	1	20:40	20:41	0:01					
07‐07‐22	2	20:49	20:50	0:01					
07‐07‐22	3	20:58	21:22	0:24					
08‐07‐22	4	22:01	22:10	0:09					
08‐07‐22	5	0:40	1:52	1:12					
08‐07‐22	6	2:37	2:40	0:03					
08‐07‐22	1	20:46	20:56	0:10					
08‐07‐22	2	21:13	21:16	0:03					
08‐07‐22	3	21:31	22:10	0:39					
08‐07‐22	4	22:50	23:37	0:47					
09‐07‐22	5	1:14	2:12	0:58					
09‐07‐22	1	21:25	22:22	0:57					
09‐07‐22	2	23:34	1:40	2:06					
09‐07‐22	3	2:43	2:44	0:01					
10‐07‐22	1	21:19	21:20	0:01	10‐07‐22	1	20:46	20:58	0:12
11‐07‐22	2	0:01	0:40	0:39	10‐07‐22	2	22:04	22:46	0:42
11‐07‐22	1	22:10	23:20	1:10	11‐07‐22	1	20:28	20:29	0:01
12‐07‐22	2	0:07	1:40	1:33	11‐07‐22	2	20:37	20:38	0:01
					12‐07‐22	3	2:37	2:43	0:06
12‐07‐22	1	20:37	20:38	0:01	12‐07‐22	1	20:46	20:52	0:06
12‐07‐22	2	22:55	23:07	0:12	12‐07‐22	2	21:16	21:58	0:42
13‐07‐22	3	2:10	2:25	0:15	12‐07‐22	3	23:43	0:46	1:03
13‐07‐22	1	22:22	23:40	1:18	13‐07‐22	1	20:25	20:26	0:01
					13‐07‐22	2	21:01	21:04	0:03
					14‐07‐22	3	0:43	1:39	0:56
					14‐07‐22	4	2:10	2:25	0:15
15‐07‐22	1	0:13	0:49	0:36	14‐07‐22	1	20:46	20:55	0:09
					14‐07‐22	2	21:34	22:22	0:48
					15‐07‐22	1	19:58	20:07	0:09
					15‐07‐22	2	20:22	Pred	

*Note:* Visit = the number of visits per night, start = time of arrival at nest, stop = time of departure from the nest, duration = the total minimum duration of attendance in minutes (given that GPS data had an interval of 3 min). Times are UTC times.

During the few days, we obtained synchronous GPS‐data of the male and the two females, we observed that male I96 attended both nests when the respective female departed on a foraging flight. For example, on 4 July 2022 (partial data due to deployment of the logger), male I96 incubated the eggs of the eastern nest (female I95) until she returned from foraging. Afterwards, he immediately flew towards the western nest (female M042), where he relieved the female from incubating. He did not wait for M042 to return from foraging, but left the eggs 10 min before her return. During the second night (5 July; Figure 3), the male first visited the western nest, but M042 had already left the nest 10 min earlier. The male incubated for only 10 min and it took another 45 min before M042 returned. After his departure at the nest of M042, the male immediately flew towards the eastern nest where he relieved female I95. In the next hours, the male and his primary female relieved each other three times from brooding freshly hatched chicks, such that they were never left unattended. On the third night (6 July) male I96 relieved female M042 from incubating once and did not leave the eggs unattended (no data from female I95 were available for that night).

## 3 Discussion

Using GPS‐data and accelerometer data, we provide first insights into the division of parental care activities of a male nightjar (I96) that formed a pair with two females whose nests were approximately 150 m apart. The two clutches were started approximately 1 week apart. Compared to a previous report on social polygyny, the two broods of this polygynous male were further apart in distance (±40 m) (Cleere and Nurney 1998), but closer in time (±14 days) (Cleere and Nurney 1998). Based on the difference in laying date, the difference in effort invested by the male in both nests, the fact that female I95 already bred with the male since 2018 and that he only sang in proximity of the nest of I95, we conclude that I95 was the primary female, and that the inexperienced, yearling female M042 was the secondary female of this polygynous trio.

Our data suggest that the male assisted the females during incubation, as well as during the nestling period (presumably by feeding and brooding the chicks). Typically, when the male came to the nest, the primary and/or secondary female went to the foraging areas. Our data further show that the secondary female received less assistance during incubation than the primary female and that her eggs were often left unattended. Such reduced assistance during incubation has also been observed in other socially polygynous species with biparental care (e.g., Lundberg and Alatalo 1992; Pinxten and Eens 1994). Thus, the eggs of the unattended nest need to be rewarmed more frequently, presumably increasing the metabolic costs to the secondary female (Biebach 1979). Once the chicks of the secondary female hatched, male assistance suddenly increased, presumably at a cost to the primary female. Our data only provide information on presence at the nest during part of the nestling period, so we cannot assess whether the male reduced the feeding rate at the primary brood in favour of the secondary brood (Lifjeld and Slagsvold 1991), although this is to be expected (Pinxten and Eens 1994).

In most facultative polygynous species with male parental care, the male predominantly or exclusively assists the primary female (Huk and Winkel 2006). Previous studies on several polygynous species showed that the division of male care depends on the difference in the timing of the two nests, with an increase in male care at the secondary nest when the hatching interval between the primary and secondary nests becomes smaller (e.g., Kempenaers, Verheyen, and Dhondt 1995). Here, the hatching interval was approximately 7 days and the male contributed to both nests, which fits with observations on other species.

This is only the second report on social polygyny in the European Nightjar. Because of the low detectability of nests and the general absence of population‐wide paternity studies, the frequency of facultative social polygyny might be underestimated in this species. Although we do not have direct observational data that may shed light on why this polygynous event occurred, we briefly discuss several non‐mutually exclusive explanations.

First, the area where both nests were found usually contains two or three territories of socially monogamous pairs. Yet, male density at the study site was lower than that observed in previous seasons. In 2022, the one male that was present might have had a larger territory at his disposal (Walseng et al. 2022). Alternatively, the male might have become polygynous after taking over a female that was initially paired to a neighbouring territory holder that then died (e.g., Schlicht and Kempenaers 2021). This hypothesis is supported by the absence of any song activity of the male near the secondary nest. Depending on when the take‐over occurred (if it did) relative to the female fertile period, the initial male might have sired at least one or all offspring in the brood. Unfortunately, the nest was predated before we could obtain DNA samples from the nestlings to investigate paternity.

Second, this case of facultative social polygyny may have arisen because an inexperienced female mated with an already paired male, either because she was initially not aware of the mating status of the male (e.g., if he displayed away from his primary female) or because the female could not find an unpaired male or because mating with this paired male was a better option than mating with another unpaired male (compensation for reduced care via high male quality or high territory quality; she shared the same foraging site with the male, Figure 4B). The secondary female was a second‐calendar year bird, and young birds usually arrive later at the breeding sites than older, more experienced adults (R. Evens and M. Lathouwers, personal observation). Late‐arriving inexperienced females are under time constraints to mate and may thus more easily be deceived by a male courting away from the primary female, or may have difficulties finding an unpaired male.

In summary, we described a case of facultative polygyny and quantified the time allocation (as a proxy for parental investment) by a polygynous male nightjar. The breeding ecology and mating behaviour of this crepuscular bird species remains little understood. Future studies, using a variety of methods including parentage analysis, will help shed light on the prevalence of social polygyny in nightjars, which is essential to make accurate population estimates, and on the causes and consequences of this mating system.

## Author Contributions

**Ruben Evens:** conceptualization (lead), data curation (equal), formal analysis (equal), investigation (equal), methodology (lead), supervision (equal), writing – original draft (lead), writing – review and editing (equal). **Michiel Lathouwers:** conceptualization (equal), data curation (equal), methodology (equal), validation (equal), writing – review and editing (equal). **Jitse Creemers:** conceptualization (equal), methodology (equal), visualization (equal), writing – review and editing (equal). **Eddy Ulenaers:** conceptualization (equal), resources (equal), writing – review and editing (equal). **Marcel Eens:** conceptualization (equal), funding acquisition (equal), supervision (equal), writing – review and editing (equal). **Bart Kempenaers:** conceptualization (equal), methodology (equal), resources (equal), validation (equal), writing – review and editing (lead).

## Conflicts of Interest

The authors declare no conflicts of interest.

## Data Availability

All data and code are available from the OSF Repository at: https://osf.io/3x7mv.
